# Presence of IgG Anti-gp160/120 Antibodies Confers Higher HIV Capture Capacity to Erythrocytes from HIV-Positive Individuals

**DOI:** 10.1371/journal.pone.0045808

**Published:** 2012-09-25

**Authors:** Maria Noé Garcia, Maria Sol dos Ramos Farias, Lucia Fazzi, Daniel Grasso, Roberto Daniel Rabinovich, Maria Mercedes Ávila

**Affiliations:** 1 National Reference Center for AIDS, Microbiology Department, School of Medicine, University of Buenos Aires, Buenos Aires, Argentina; 2 Department of Pathophysiology, School of Pharmacy and Biochemistry, University of Buenos Aires, Buenos Aires, Argentina; Institute of Infectious Diseases and Molecular Medicine, South Africa

## Abstract

**Background:**

HIV binding has been demonstrated in erythrocytes from HIV-positive and HIV-negative individuals. However, the presence of immunoglobulins G anti-HIV (IgG anti-HIV) in erythrocytes from HIV-positive individuals is still to be elucidated. Moreover, the capacity of erythrocytes from HIV-positive individuals to capture an additional amount of HIV has not been studied. Indeed, it is unknown if HIV binding to erythrocytes in HIV-positive persons could have consequences on the cell-free infectious virus available.

**Methodology/Principal Findings:**

IgGs anti-HIV associated to erythrocytes were found in 77.3% (58/75) of the HIV-positive individuals studied and the IgGs anti-gp160 and anti-p24 were the most frequently found. We found a positive association between detectable plasma viral load (pVL) and presence of IgGs anti-HIV associated to erythrocyte (*p*<0.005), though the anti-p24/160 were present with or without detectable pVL. The HIV capture capacity was higher in erythrocytes from HIV-positive than HIV-negative individuals (*p*<0.0001). Furthermore, among the HIV-positive individuals the higher viral capture capacity was associated with the presence of anti-gp160/gp120 on erythrocytes. Moreover, the viral capture by erythrocytes was independent of pVL (rho = 0.022, *p* = 0.8817). Additionally, reduction of cell-free infectious virus and available viral load was observed in the presence of erythrocytes from HIV-positive individuals.

**Conclusions/Significance:**

Results suggest that in HIV-positive individuals, erythrocytes are capable of capturing high amounts of HIV by the presence of IgGs anti-gp160/120 on their membranes and this may produce a reduction in the available free virus. Finally, the current measurement of pVL would underestimate the real viral quantity due to the HIV binding through specific antibodies to erythrocytes.

## Introduction

During persistent chronic viral infections, antibody and virus production occurs simultaneously, creating conditions for immune complexes formation and the subsequent adherence to erythrocytes (immune-adherence) [1], [2]. Immune complexes, namely antigens opsonized by antibodies and complement, are bound to erythrocytes through specific interactions of the complement protein C3b with the erythrocyte complement receptor 1 (CR1 or CD35). Eventually, erythrocytes carried in the bloodstream pass through the spleen and liver where macrophages take up the immune complexes [3], [4].Human immunodeficiency virus type 1 (HIV) particles are known to be capable of binding to erythrocytes in HIV-negative individuals *in vitro* by at least three mechanisms: 1) binding of immune complexes through the CR1 receptor, 2) binding of HIV to CR1 by complement proteins but in absence of antibodies, and 3) direct binding of HIV to Duffy antigen receptor for chemokines (CD55 or DARC) present on erythrocytes [5]–[10].

Recently we have shown the presence of HIV viral load and p24-antigen on erythrocytes from HIV-positive individuals even in patients with undetectable plasma viral load (pVL) [1]. In that study, presence of p24-antigen was found in more than 70% of the patients with detectable pVL and in some patients with undetectable pVL [1]. Moreover, Hess *et al*., 2002 [11] reported detectable HIV viral loads in purified erythrocytes from a group of patients under highly active antiretroviral therapy for a long period of time.

Several authors have suggested that erythrocyte-bound HIV might comprise an important surface reservoir for *trans* infection of permissive cells [9]–[13]. Furthermore, it has been demonstrated that HIV infects CD4-positive cells approximately 100-fold more efficiently when it is associated to erythrocyte than when it is present as cell free viral particles [9], [10]. Besides, the virus bound to erythrocytes may be less sensitive to neutralization mediated by some specific antibodies [14]. Altogether, these data highlight the relevance in understanding the HIV-erythrocyte interaction during the HIV pathogenesis.

One of the proposed mechanisms for HIV binding to erythrocytes involves immune complexes [5]–[7], [13]. However, the presence and pattern of immunoglobulins G anti HIV (IgG anti-HIV) in erythrocytes from HIV-positive individuals is still to be demonstrated. Moreover, in spite that erythrocytes are virus carriers, the capacity of erythrocytes from HIV-positive individuals to attach virus and/or antigen at the cell surface has not been studied. Indeed, it is unknown if HIV binding to erythrocytes of HIV-positive individuals could quantitatively affect the cell-free infectious virus available.

In this study we demonstrate the presence of IgGs anti-HIV associated to erythrocytes from HIV-positive individuals. Interestingly, we found that erythrocytes from HIV-positive individuals have higher capacity of viral capture than erythrocytes from HIV-negative individuals. Furthermore, this higher capacity was associated with the presence of the IgG anti-gp160/gp120 in erythrocytes. Finally, erythrocytes quantitatively decrease the available cell-free infectious virus.

## Results

### IgGs Anti-HIV are Present on Erythrocytes from HIV-positive Individuals

In order to investigate the presence and pattern of IgGs anti-HIV in erythrocytes from HIV-positive individuals, blood samples of 75 individuals were evaluated. IgGs anti-HIV were determined by western blot assay in: purified erythrocytes (IgG anti-HIV-E), supernatant of the last erythrocytes washing (IgG anti-HIV-W) and plasma (IgG anti-HIV-P). One or more IgG anti-HIV-E antibodies were found in 77.3% (58/75) of the studied individuals. IgGs anti-HIV antibodies most frequently associated to erythrocytes were anti-gp160 in 84.5% (49/58), anti-p24 in 63.8% (37/58), anti-p34 in 39.6% (23/58), anti-p68 in 34.5% (20/58), anti-gp41 in 25.8% (15/58), anti-p55 in 22.4% (13/58), anti-gp120 in 18.9% (11/58), anti-p52 in 13.8% (8/58), anti-p40 in 6.9% (4/58) and anti-p18 in 1.7% (1/58) (**Table S1**). Anti-gp41 and anti-gp120 antibodies were found in those samples where anti-gp160 was also detectable. In contrast, presence of anti-gp160 was not always accompanied by presence of anti-gp120 and/or anti-gp41 (**Table S1**).

Consecutively, the association between pVL and presence of IgG anti-HIV-E was studied. To accomplish this objective, pVL was determined in blood samples of the 75 individuals listed above. Only 14 out of 25 individuals, with undetectable pVL (<50 copies per ml), presented IgG anti-HIV-E. On the contrary, IgG anti-HIV-E were detected in 44 out of 50 individuals that presented detectable pVL (≥50 copies per ml) and a significant positive relationship between detectable pVL and the presence of IgG anti-HIV-E was found (*p*<0.005). Anti-p24 and anti-gp160 antibodies were present on erythrocytes in patients with or without detectable pVL. However, all the other antibodies mentioned above were found only among individuals with detectable pVL. Moreover, a significant positive relationship was observed between detectable pVL and presence of anti-gp160 (*p*<0.05), anti-gp120 (*p*<0.05), anti-p68 (*p*<0.0005), anti-p55 (*p*<0.005), anti-p52 (*p*<0.05), anti-gp41 (*p*<0.005) and anti-gp34 (*p*<0.0005) in erythrocytes. No significant relationship was found for anti-p40, anti-p24 and anti-p18 (**Table S1**).

**Figure 1:**
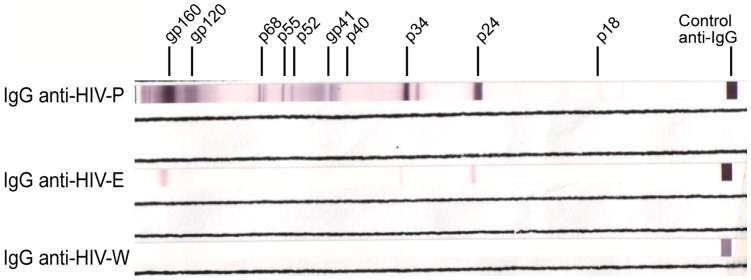
Anti-HIV antibodies pattern associated to erythrocytes. IgG anti-HIV were determined by western blot in: plasma (IgG anti-HIV-P), purified erythrocytes (IgG anti-HIV-E), and supernatant of the last wash of erythrocytes purification process (IgG anti-HIV-W). Figure shows a representative pattern of IgG anti-HIV in erythrocytes from HIV-positive individuals. Presence of IgG anti-gp160, IgG anti-p24 and IgG anti-p34 in erythrocytes of one patient is observed.

While all IgGs anti-HIV were detected in plasma, the predominant pattern on erythrocytes was anti-gp160 and/or anti-p24. It is important to highlight, that no IgG anti-HIV was ever detected in the supernatant of the last erythrocyte washing (IgG anti-HIV-W). This last demonstrates that purified erythrocytes did not contain contaminating IgG anti-HIV from other blood fractions such as plasma (**Figure 1**).

Since different IgG anti-HIV patterns were found in plasma and erythrocytes from the same patients, we evaluated the selectivity of IgG anti-HIV adherence to erythrocyte. To accomplish this goal, we compared the IgG anti-HIV pattern in plasma and erythrocytes at the same concentration in eight samples from HIV-positive individuals (see materials and methods section). Notably, we could not found a correspondence between IgG anti-HIV pattern in erythrocytes and plasmas at same concentration of IgGs (**Figure S1**). Altogether, these results demonstrate the existence of a selective IgG anti-HIV adherence to erythrocytes of HIV-positive individuals.

### p24-antigen Detection on Erythrocytes is Accompanied by Presence of IgG Anti-HIV

Recently, we have demonstrated the presence of p24-antigen in erythrocytes from HIV-positive individuals [1]. Therefore, in order to determine if detection of p24-antigen in erythrocytes (Ag-E) implies presence of IgGs anti-HIV-E, the pattern of antibodies present on erythrocytes was determined in samples of 12 individuals who had detectable Ag-E. We found that all of these individuals presented at least one IgG anti-HIV in erythrocytes. Determinations showed presence of anti-gp160 in 11 out of the 12 individuals, 6 presented IgG anti-p24 and 6 IgG anti-p34 (**Table S1**). Although, there are not enough determinations for a statistical analysis, these results suggest a possible association between detection of Ag-E and presence of IgG anti-HIV on erythrocyte membrane.

### Erythrocytes from HIV-positive Individuals Possess Higher HIV Capture Capacity than Erythrocytes from HIV-negative Individuals

Erythrocytes are in high number providing a large surface in the bloodstream. Besides, presence of HIV-RNA [1], [11], p24-antigen [1] and IgG anti-HIV was shown on erythrocytes from HIV-positive individuals. In order to establish whether erythrocytes from HIV-positive individuals are still capable to attach additional virus and/or antigen at the cell surface, we analyzed HIV capture by erythrocytes, pVL and IgG anti HIV-E in 48 blood samples from HIV-positive (48 of the 75 belonging to Table S1) and 15 from HIV-negative individuals.

**Figure 2:**
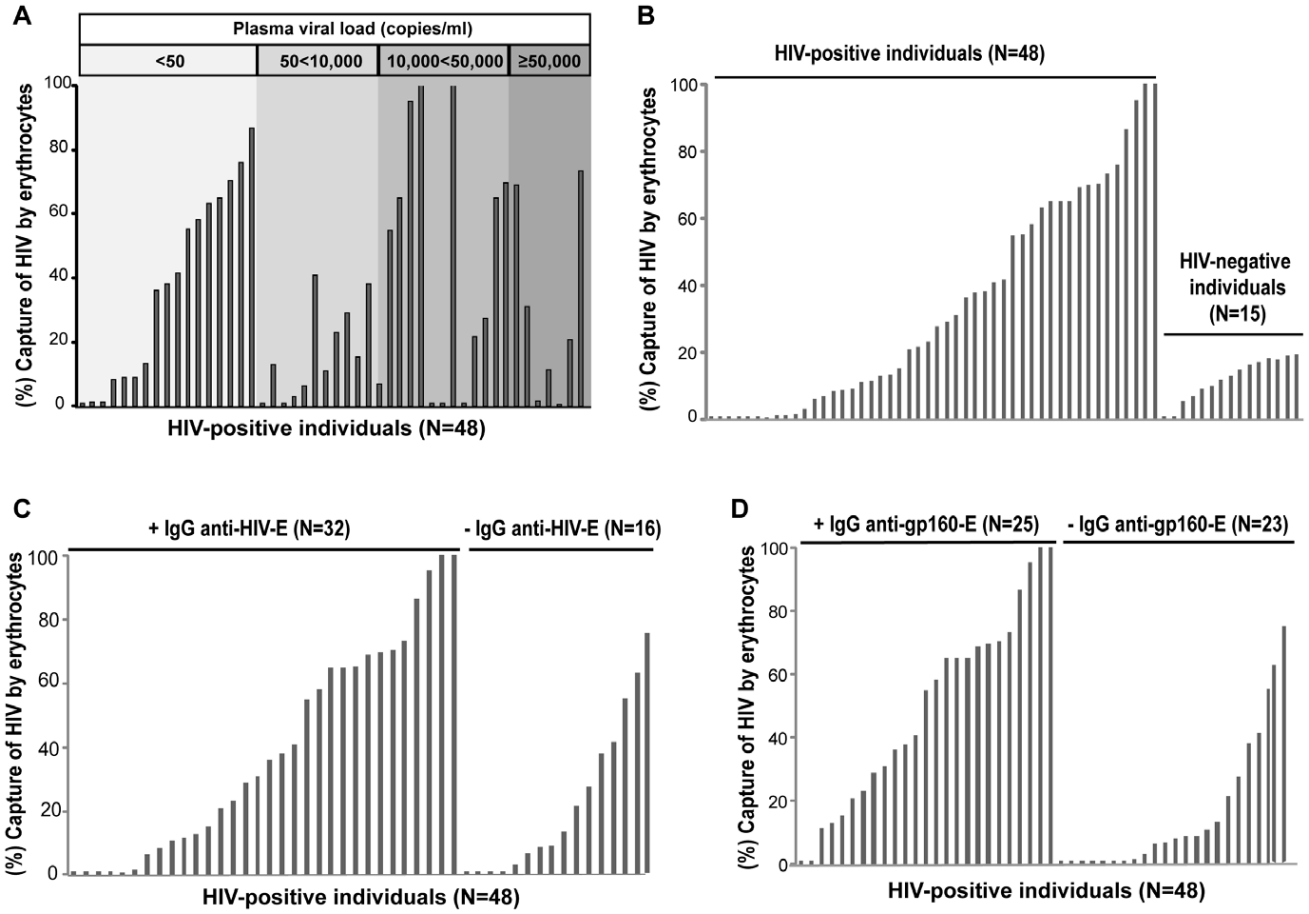
Capture of HIV by erythrocytes from HIV-positive individuals. Plots show capture of HIV by erythrocytes (percentage of p24-antigen bound to erythrocytes, viral capture assay) from 48 HIV-positive individuals (48 of 75 belonging to Table S1) and 15 HIV-negative individuals. Each bar represents an individual. (A) HIV capture by erythrocytes according to plasma viral load. Range of plasma viral load is indicated at the top. Individuals are located according to the value of plasma viral load at the time of study. No correlation is observed between the ability of HIV capture by erythrocytes and plasma viral load of patients (rho = 0.022, *p* = 0.8817, correlation was evaluated using Spearman’s rank correlation), demonstrating that viral capture by erythrocytes occurs independently of plasma viral load. (B) HIV capture by erythrocytes from 48 HIV-positive and 15 HIV-negative individuals. Individuals are located in increasing values of viral capture capacity. Both groups show a significant difference between mean levels of HIV capture (*p*<0.0001). (C) The 48 HIV-positive individuals are grouped according to the presence (+ IgG anti-HIV-E, N = 32) or absence (- IgG anti-HIV-E, N = 16) of at least one IgG anti-HIV associated to erythrocytes. No statistical difference was found between presence of IgG anti-HIV-E and a viral capture >20% by erythrocytes (*p*>0.5). (D) The 48 HIV-positive individuals are grouped according to the presence (+ IgG anti-gp160-E, N = 25) or absence (- IgG anti-gp160-E, N = 23) of IgG anti-gp160 associated to erythrocytes. Erythrocytes with presence of IgG anti-gp160 show an HIV capture higher than 20% (*p*<0.0005).

In erythrocytes from HIV-positive individuals an HIV capture higher than 20% was found in the 56% (27/48) of the samples with a mean of 33.82% (**Figure 2** **A and B**, **Table S1**). No correlation was observed between the HIV capture capacity by erythrocytes and the pVL value (rho = 0.022, *p* = 0.8817). This result demonstrates that the erythrocyte viral capture occurs independently of the pVL (**Figure 2A**). In contrast, the HIV capture capacity by erythrocytes from HIV-negative individuals was less than 20% in all cases, with a mean of 11.95% (**Figure 2B**). Analyzing both groups, a significant difference was found between the means of HIV capture capacity by erythrocytes in each group (*p*<0.0001). These results suggest that erythrocytes, in HIV-positive individuals, not only are in circulation with HIV/antigen bound in their membranes, but are also able to capture an additional amount of virus.

### The IgG Anti-gp160/120 is Responsible for the Higher HIV Capture Capacity of Erythrocytes from HIV-positive Individuals

Since erythrocytes from HIV-positive individuals possess higher HIV capture capacity we wondered if this fact is related to the presence of IgGs anti-HIV on erythrocytes. In this regard, we could not find a relationship between the presence of IgG anti-HIV-E and a HIV capture capacity higher than 20% (*p*>0.5) (**Figure 2C**). Indeed, we did not find a statistical relationship between the HIV capture capacity mean level and the presence of IgG anti-HIV-E (*p*>0.05) (**Figure 2C**). Nevertheless, there is an extremely significant difference between the means of HIV capture capacity in individuals with (N = 25, mean 49.22%) and without (N = 23, mean 17.11%) IgG anti-gp160 associated to erythrocyte (*p*<0.0005) (**Figure 2D**). From those patients with IgG anti-gp160, 20 out of 25 presented an HIV capture capacity higher than 20% and 13 higher than 50% (**Figure 2D**). On the other hand, from the 23 individuals without IgG anti-gp160, only 7 presented values higher than 20% and 3 over 50% (**Figure 2D**). These data strongly suggest that, in erythrocytes from HIV-positive individuals, exist a positive association between presence of IgG anti-gp160 and HIV capture capacity.

**Figure 3:**
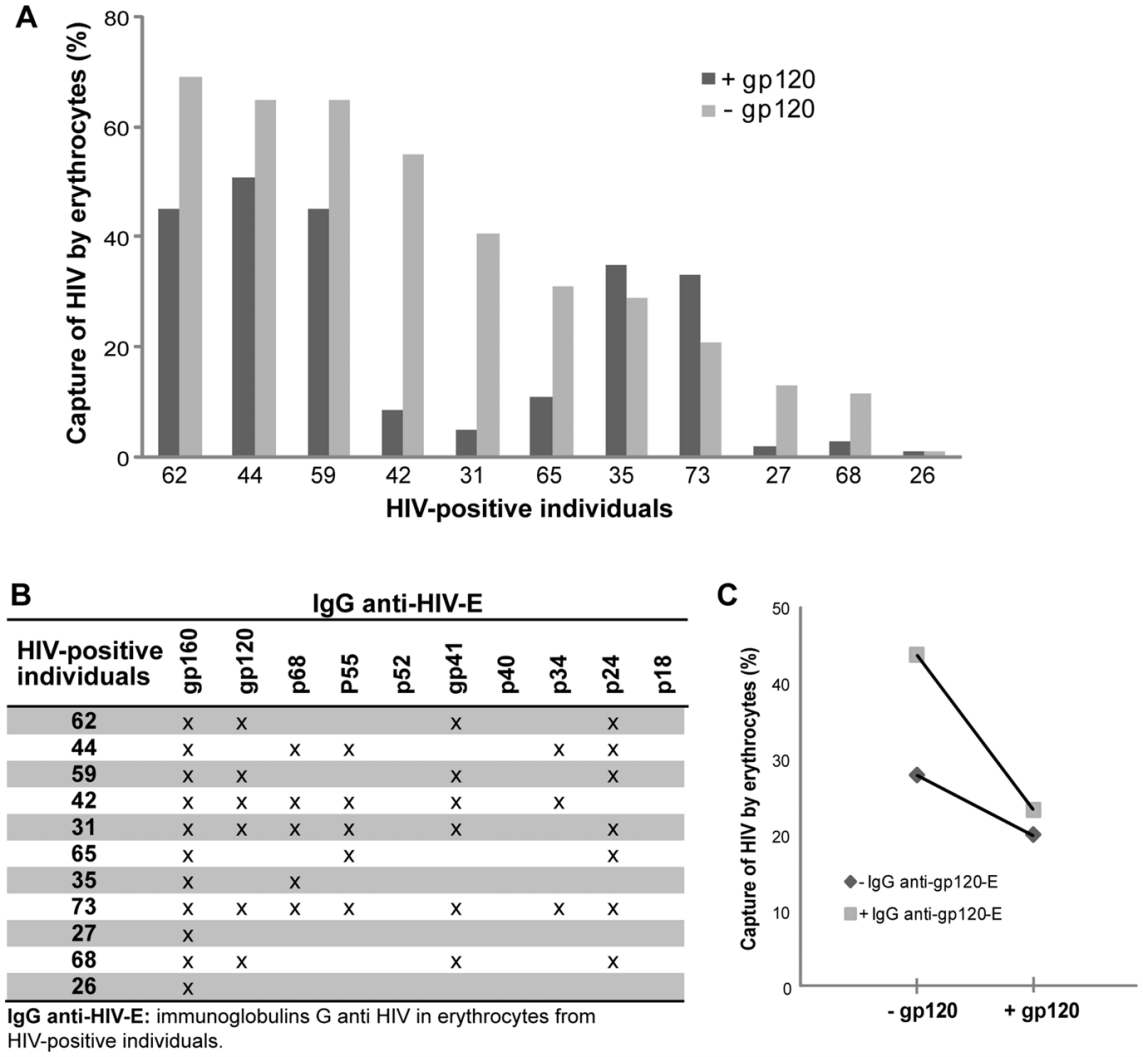
Inhibition of HIV capture by recombinant gp120. (**A**) Percentage of HIV capture by erythrocytes from HIV-positive individuals (11 of 75 belonging to Table S1). Erythrocytes were previously incubated with soluble gp120 (Inhibition of viral capture assay, dark gray bars) or without gp120 (viral capture assay, light gray bars). HIV capture by erythrocytes was inhibited by viral gp120 (*p*<0.05) and the variation between individuals was significant (*p*<0.05) (two-way ANOVA with random effects). This result indicates that the large-scale inhibitory effect varies according to the subject from whom erythrocytes were obtained. (**B**) The table lists IgG anti-HIV antibodies present in the erythrocytes of the 11 individuals. All of them have IgG anti-gp160, and 6 individuals with IgG anti-gp120 also present IgG anti-gp41 on their erythrocytes (patients 62, 59, 42, 31, 71 and 68). (**C**) The mean percentage of viral capture of HIV by erythrocytes with or without inhibition with soluble gp120 is plotted in individuals who present IgG anti-gp120 in erythrocytes (+ IgG anti-gp120-E, line with square) and in individuals who do not present IgG anti-gp120 in erythrocytes (- IgG anti-gp120-E, line with rhomb). The inhibitory effect mainly occurs in individuals with IgG anti-gp120 on their erythrocytes (*p*<0.05, repeated measures analysis).

The gp160 antigen is a precursor that is cleaved in order to form the gp120 and gp41 proteins. Therefore, these three proteins share several epitopes. To elucidate whether HIV capture by erythrocytes relies on the IgG anti-gp120, an inhibition assay of the viral capture was performed in erythrocytes from 11 HIV-positive individuals (in all cases the IgG anti HIV-E pattern was determined prior to viral capture assay). **Figure 3A** shows that incubation of erythrocytes with recombinant gp120 protein decreases the HIV capture (*p*<0.05, two-way ANOVA with random effects). Moreover, the inhibitory effect varied according to the subject from whom erythrocytes were obtained (*p*<0.05, two-way ANOVA with random effects). Since erythrocytes with IgG anti-gp120 presented higher viral capture, the inhibitory effect was markedly more important in this group than in the group of erythrocytes without IgG anti-gp120 (*p*<0.05, repeated measures analysis) (**Figure 3** **B and C**). Presented data only shows the results obtained for gp120 IIIB, although similar results were obtained with the MN and CM variants (data not shown). In summary, higher HIV capture is associated to individuals with IgG anti-gp120 on their erythrocytes.

### Loss of HIV Infectivity and Viral Load is due to the Presence of Erythrocytes

Given that it has been demonstrated that erythrocytes are capable of capturing HIV, we wondered whether erythrocytes may affect HIV infectivity in presence of antibodies from HIV-positive individuals. In order to address this issue, plasma dilutions from 14 HIV-positive individuals were used as source of HIV-specific antibodies and then incubated with CXCR4-tropic strains of HIV IIIB.

**Figure 4:**
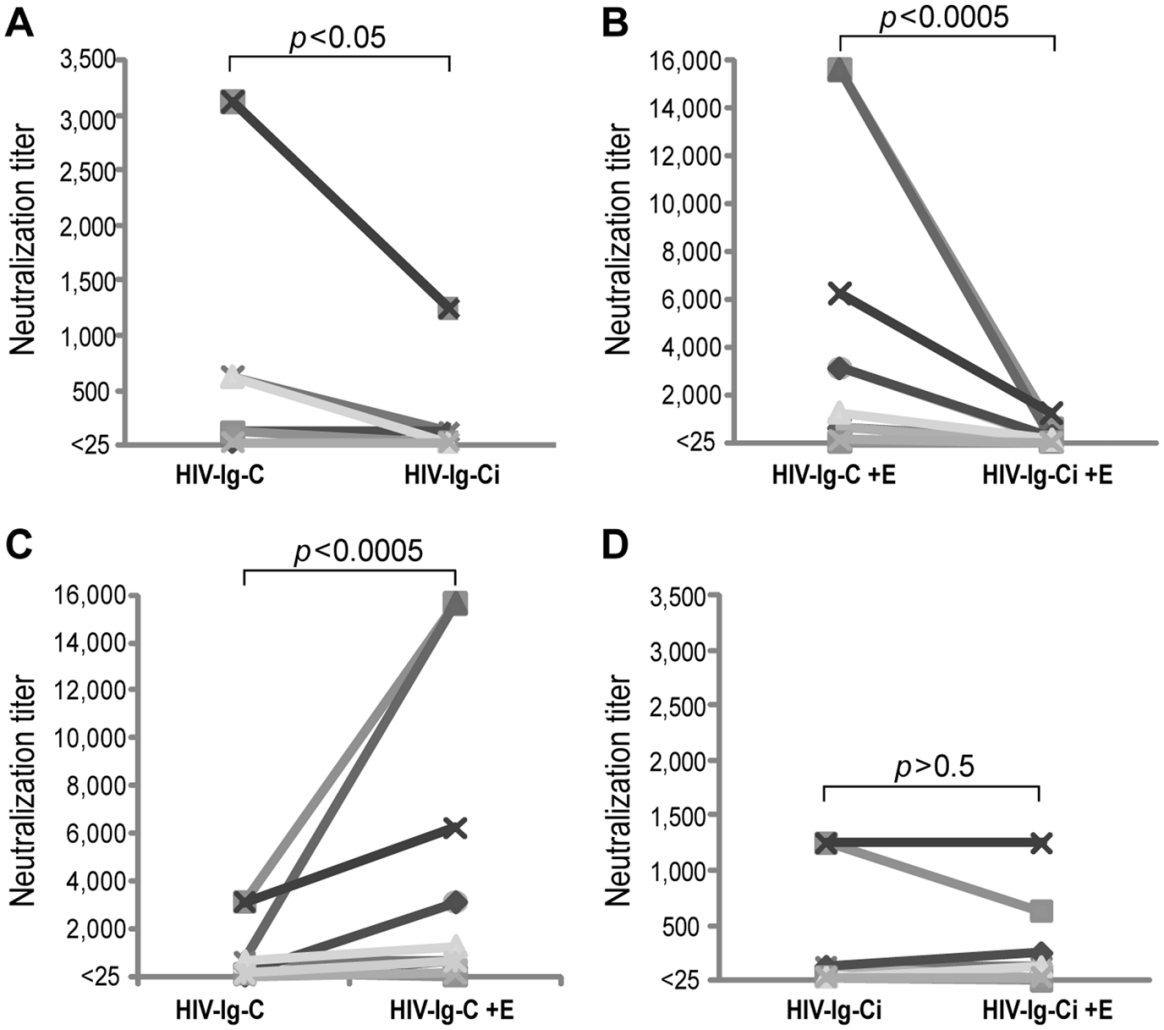
Presence of erythrocytes contribute to the loss of HIV infectivity. Figure shows neutralization titers, expressed as the inverse of the highest dilution that is able to inhibit syncytia formation (loss of infectivity) in MT-2 cells. Plasma dilutions from 14 HIV-positives individuals (Ig) (as a source of HIV-specific antibodies) were incubated with CXCR4-tropic strain of HIV IIIB (HIV) in presence of: normal human serum as source of complement (C) (HIV + Ig + C) or inactivated normal human serum (Ci) (HIV + Ig + Ci). Each of these was exposed to erythrocytes from HIV-negative individuals (E) (HIV-Ig-C +E or HIV-Ig-Ci + E). The remaining infectivity in supernatants of each incubation was measured. Data points are means of three independent experiments with plasma from the same patient. (**A**) In the absence of erythrocytes, the loss of HIV infectivity in plasma was significantly greater when complement was present than when it was inactivated (*p*<0.05, Wilcoxon signed-rank test). (**B**) The presence of erythrocytes incubated with complement led to a loss of HIV infectivity considered extremely significant when compared to erythrocytes incubated with inactivated complement (*p*<0.0005, Wilcoxon signed-rank test). (**C**) Loss of infectivity in the presence of complement was exacerbated when erythrocytes were also present (*p*<0.0005 Wilcoxon signed-rank test). (**D**) The loss of infectivity in the presence of inactivated complement was not affected by the presence or absence of erythrocytes (*p*>0.5 Wilcoxon signed-rank test). Results demonstrate that the loss of HIV infectivity is increased *in vitro* in an environment where erythrocytes are present.

In absence of erythrocytes, the reduction of HIV infectivity in plasma was significantly greater when complement was present than when it was inactivated (*p*<0.05, Wilcoxon signed-rank test, mean 678 vs 244), as reported by Huber *et al.,* 2006 [15] (**Figure 4A**). The presence of erythrocytes, concomitantly incubated with complement, led to a loss of HIV infectivity considered extremely significant compared to erythrocytes incubated with inactivated complement (mean 3,675 vs. 210, *p*<0.0005, Wilcoxon signed-rank test) **Figure 4B**. More importantly, the loss of HIV infectivity in presence of complement was exacerbated when erythrocytes were also present (639 vs. 3,457, *p*<0.0005 Wilcoxon signed-rank test) (**Figure 4C**). On the other hand, when complement was inactivated, the loss of HIV infectivity was not affected by the presence or the absence of erythrocytes (mean 210 vs. 244, *p*>0.5 Wilcoxon signed-rank test) and it was always lower than when the non-inactivated complement was present (**Figure 4D**).

**Figure 5:**
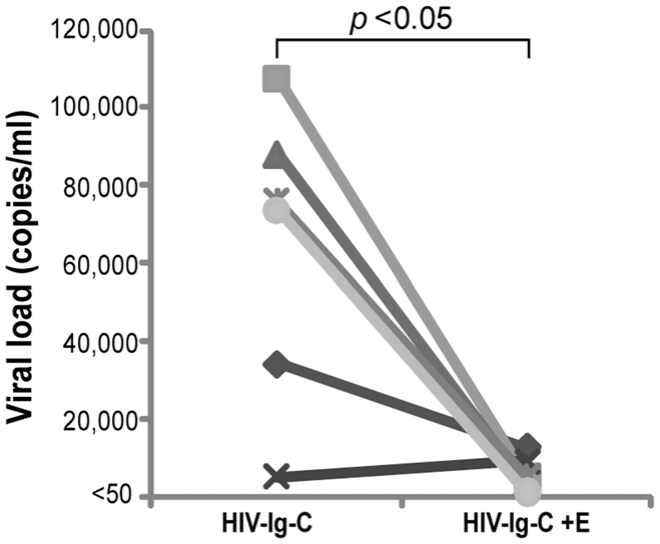
Loss of viral load by presence of erythrocytes. Figure shows viral load (copies/ml) of plasmas from 6 HIV-positive individuals (Ig), as a source of HIV-specific antibodies, incubated with HIV IIIB (HIV) in presence of normal human serum as complement source (C) and in presence (+E) or absence of erythrocytes. Viral load was determined in each supernatant. Data points are means of three independent experiments with plasma from the same patient. The difference between the value of viral load in presence and absence of erythrocytes was compared using Wilcoxon signed-rank test.

In another series of experiments, the presence of erythrocytes accompanied of immunoglobulins anti-HIV and complement, was sufficient to produce a significant decrease in HIV viral load (viral particle) (*p*<0.05 Wilcoxon signed-rank test) (**Figure 5**).

These results demonstrate that complement and erythrocytes play a key role in the HIV infectivity and suggest that erythrocyte-mediated sequestering of HIV particles may be relevant to the plasma viral load of patients.

### The Presence of Erythrocytes from HIV-positive Individuals Produces Loss of HIV Infectivity

We have demonstrated the ability of erythrocytes from HIV-positive individuals to capture free HIV. This remarkable property is complemented by the fact that erythrocytes from HIV-negative individuals are able to decrease the viral load and HIV infectivity. Those results led us to study the ability of erythrocytes from HIV-positive individuals to reduce the HIV infectivity. With that purpose, erythrocytes from HIV-positive individuals were incubated with a known amount of virus in a similar way as that it was explained in the last section but in absence of immunoglobulins anti-HIV and complement. **Figure 6** shows that HIV incubation with erythrocytes from HIV-positive individuals significantly decreased the HIV titer in the medium (*p*<0.001). The erythrocytes from HIV-negative individuals also reduced the HIV titer (*p*<0.01) and despite that this reduction looks lower than data from HIV-positive individuals, they were not statistically different (*p*>0.05) (**Figure 6**).

**Figure:**
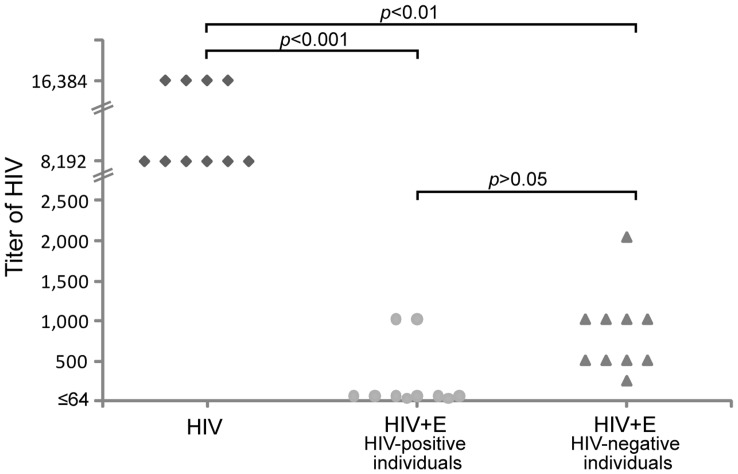
Loss of HIV infectivity by presence of erythrocytes from HIV-positive individuals. HIV IIIB (HIV) was incubated with erythrocytes from HIV-positive (HIV + E _HIV-positive individuals_) or HIV-negative (HIV + E _HIV-negative individuals_) individuals. The figure shows the titers of infectious HIV in each supernatant. A significant reduction in the titer of HIV is observed in the presence of erythrocytes from HIV-positive (*p*<0.001) or HIV-negative individuals (*p*<0.01). No statistical difference is observed in HIV titre reduction between erythrocytes from HIV-positive versus HIV-negative individuals (*p*>0.05). Differences were compared using Kruskal-Wallis Test.

## Discussion

The present study showed that IgG anti-HIV were associated to erythrocytes in 77.3% (58/75) of HIV-positive individuals suggesting that viral adherence through antibodies may occur *in vivo*. The most frequently associated to erythrocytes was anti-gp160, followed by anti-p24. The presence of IgG anti-gp120 or IgG anti-gp160 in erythrocytes may reflect the presence of erythrocyte-associated immune complexes formed by viral particles and/or soluble antigens. Though, p24-antigen may be not associated to viral particles in plasma [16]–[18] IgG anti-p24 associated to erythrocytes could be present even in individuals with undetectable viral loads.

A detectable pVL indicates that viral particles are circulating where the virus could be recognized and coated by specific antibodies and complement proteins to finally bind to erythrocytes. In this regard the association between the presence of IgGs anti-HIV on erythrocytes and detectable pVL suggests the presence of viral particles and/or viral antigens on erythrocytes. More significant is the association between the presence of IgG anti-gp160 and detectable pVL because it would reflect the presence of viral particles associated to erythrocytes through immune-complexes. On the other hand, the presence of IgG anti-p24 was not associated to detectable pVL. Indeed the erythrocyte-associated p24 antigen is not associated to detectable pVL [1]. This coincidence is striking due to the fact that in both cases it seems that detectable pVL might not be related to p24-antigen or IgG anti-p24 in erythrocytes.

Altogether, the presence of IgG anti-HIV in erythrocytes from HIV-positive individuals, supported by previous data about p24-antigen [1] and HIV-RNA [1], [11] detected in erythrocytes, suggests that virus and/or viral antigens bind to erythrocytes by immunoadherence in HIV-positive individuals.

Only 14 patients of the studied population presented IgG anti-HIV on erythrocytes with undetectable pVL (**Table S1**). This result can be understood taking into account, as explained above, that it is not necessary a detectable pVL for antigen detection on erythrocytes [1]. We also observed that some individuals have had a detectable pVL without detection of IgG anti-HIV on erythrocytes (**Table S1**). This fact could indicate a different viral clearance or a viral capture independent of the presence of antibodies in erythrocytes [9], [10].

The data support that the erythrocytes from HIV-positive individuals may not only have associated RNA-HIV, p24-antigen [1], [11] and IgG anti-HIV but also bear the capacity to capture additional HIV to its membrane. The viral capture capacity assay performed on erythrocytes from a group of HIV-positive individuals showed that about 50% of them captured more than 20% of virus. Moreover, we ruled out the possibility that p24-antigen on patients’ erythrocytes interfere with the capture capacity assay. On the contrary, erythrocytes from HIV-negative individuals always capture a maximum of 19.5%. These observations differ from that observed by Beck *et al*., 2009 [10], who found that erythrocytes from non-infected individuals could capture up to 6% of HIV. This could be explained since lower quantities of virus stock enriched in particles were used in the present study.

Of note is that the HIV capture capacity by erythrocytes from HIV-positive individuals was not related to the value of pVL. Therefore, the HIV bound to erythrocytes was not in line with the circulating viral particles. This suggests that erythrocytes are not saturated *in vivo*, thus additional virus binding and virus clearance may occur simultaneously in the patients.

The presence of IgG anti-gp160 in erythrocytes yielded a statistical increase in HIV capture capacity (*p*<0.0005). When erythrocytes were blocked by soluble gp120, the viral capture decreased considerably and this effect was marked in those individuals with IgG anti-gp120 in erythrocytes (**Figure 3**). However, as the inhibition by recombinant gp120 was never completed other mechanisms such as HIV binding to erythrocytes by DARC antigen [9], [10], could be involved. Of note, the capture inhibition assay was performed with three different strains of gp120 (IIIB, MN and CM) with similar results. This suggests that gp120-mediated HIV capture inhibition may be not virus strain specific.

Note that patients 65 and 27 (**Figure 3B**) have the same western blot pattern, neither of them show presence of anti-gp120 and anti-gp41 IgGs. Nevertheless, both of them have presence of IgG anti-gp160. Since gp160 is the precursor of gp120 and gp41, a possible explanation is that such antibodies are present but they are not detected due to higher affinity to gp160.

The proposed mechanism for erythrocyte-mediated HIV capture is that the circulating virus is exposed to recognition by specific antibodies and afterwards it is attacked by the complement system. This induces the formation of circulating immune complexes, their binding to erythrocytes and posterior depuration. Those erythrocytes carrying immune complexes, with antibodies in excess, expose free paratopes which could allow additional virus capture. Consequently, the viral capture by erythrocytes in HIV-positive individuals could play an important role in the clearance of free virus and its delivery to macrophages in spleen and liver [4]. Even more, erythrocytes might bind HIV virions which are then potentially available for *trans* infection of permissive target cells.

Reduction of viral infectivity by erythrocytes has already been suggested for type 5 adenovirus (Ad5) [19]. It has been demonstrated that erythrocytes efficiently sequester Ad5, reducing its extravasation and infectivity [19]. Additionally, similar results have been reported for parvovirus B19 [20]. These facts highly support our results about significant reduction of HIV infectivity in plasma when it is incubated with erythrocytes. Circulating HIV is exposed to neutralization and virolysis by the complement system [15], in an environment where a high number of erythrocytes are capable of capturing HIV. The current study demonstrates that in the presence of antibodies and complement, erythrocytes contribute to the reduction of the viral load and HIV infectivity. Although this study was not intended to elucidate whether the erythrocyte-mediated reduction of HIV infectivity is due to direct binding of HIV to erythrocytes or to an enhanced neutralization and/or complement-mediated lysis, the viral capture assays suggest the first option where the erythrocytes play a major role.

The results presented here suggest that the current measurement of pVL in HIV-positive individuals would underestimate the real viral quantity due to the HIV binding to erythrocytes mediated by complement and/or antibodies. On the contrary, the amount of antibodies bound to erythrocytes would not alter the detection of plasma antibodies in HIV-positive patients. As it was presented in this work, the amount of IgGs present on the erythrocytes is far below to circulating IgGs in plasma. This therefore, suggests that the association of IgGs anti-HIV to erythrocytes has a relevant role in HIV availability in plasma but it is not enough to significantly modify the quantification of IgGs anti-HIV. Moreover, by enhancing HIV binding to erythrocytes, the presence of HIV antibodies could play an important role in the loss of infectivity, beyond its neutralizing capacity.

In conclusion, this study demonstrates the presence of IgG anti-HIV associated to erythrocytes from HIV-positive individuals, being the anti-gp160 and anti-p24 antibodies the most frequently found. Moreover, erythrocytes from HIV-positive individuals possess higher HIV capture capacity than erythrocytes from HIV-negative individuals, and this is associated with the presence of IgG anti-gp160/gp120 on erythrocyte membrane. In addition, erythrocytes are capable to significantly reduce HIV infectivity in plasma, but may keep infective virus bound to its membrane. Finally, future studies in the erythrocyte-HIV relationship will have a significant relevance for the understanding of HIV pathogenesis and for the follow-up of patients.

## Materials and Methods

### Study Population

A total of 117 HIV-positive individuals older than 21 years of age, who attended to the Argentinean National Reference Centre for AIDS for pVL assessment, were invited to participate in this study. The only inclusion criterion was that they must be HIV-positive. Additionally, fifty-one HIV-negative individuals were recruited as controls.

### Ethics Statement

The protocol was approved by the local Ethics Committee of Nexo AC Institutional Biomedical Review Board (IRB 00005349) and conducted in compliance with all federal regulations governing the protection of human subjects. A written informed consent was signed by each participant.

### Sample Processing

Samples were obtained by venous puncture using EDTA as anti-coagulant. Erythrocytes were processed the day of blood collection. Blood was centrifuged at 1,400 g for 10 minutes. Plasma was stored for pVL and IgG anti-HIV determination. The buffy coat was discarded. 1 ml of the erythrocyte**-**enriched fraction was diluted 1/2 in PBS, followed by addition of Dextran 4% (MW 200,000–275,000) (Biochemical), mixed by inversion and kept in vertical position for 30 minutes at RT. The erythrocyte sediment was washed four times in PBS and in each wash, supernatant and interface layers were aspirated and discarded. As control for the washing process, the supernatant of the last erythrocyte wash (IgG anti HIV-W) was taken to be used in IgG anti-HIV detection assays.

### Erythrocyte-associated Antibodies Release

Erythrocytes were subjected to acid treatment in order to release erythrocyte-associated antibodies. Briefly, 750 µl glycine-HCl buffer (pH 3.2) was added to 250 µl of purified erythrocytes at RT and centrifuged at 470 g for 5 minutes. The supernatant was neutralized to pH 7.2 with NaOH 0.1N and kept at −20°C. The glycine-HCl treatment was also performed in plasma and in supernatant of the last wash (IgG anti HIV-W) of each sample as a control of erythrocyte washing.

In order to demonstrate selectivity in adherence between antibodies and erythrocytes, we compared the IgG anti-HIV pattern in plasma and erythrocyte at same concentration in eight samples from HIV-positive individuals. The solution where IgGs are released from erythrocytes to be measured was concentrated 100-fold (IgG anti-HIV-Ec) by the use of a microconcentrator Amicon YM-10 (Millipore Corporation, Bedford, MA, USA). Less than 33.3 mg/dl of total IgGs was found in this last solution, while in plasma it ranged from 1,110 to 2,600 mg/dl. Therefore, plasma was diluted at a final concentration equal to the extent in IgG in erythrocytes (IgG anti HIV-Pd) and IgG anti-HIV were evaluated by western blot.

Measurement of IgG, IgA and IgM was made by IMMAGE Immunochemistry System (Beckman Coulter). Detection limit for IgG was <33.3 (mg/dL), for IgA it was <6.67 (mg/dL) and for IgM it was <4.17(mg/dL). Reference range for plasma is IgG 50–8,000 (mg/dL), IgA 95–385 (mg/dL) and IgM (mg/dL) 5–1,000. All studied individuals were within the normal reference ranges for IgG, IgA and IgM in plasma.

### Antibodies Pattern Determination

Antibodies pattern was determined using anti-HIV antibody test (New LAV Blot I Assay, BIO-RAD) in the following samples: plasma (IgG anti-HIV-P), purified erythrocytes (IgG anti-HIV-E) and the supernatant of the last erythrocyte washing (IgG anti HIV-W).

### Plasma Viral Load

Plasma viral load was assessed by branched DNA (b-DNA) technology, with a detection limit of 50 HIV-1 RNA copies/ml (Versant HIV-1 RNA 3.0, Bayer Co., Tarrytown, NY).

### p24-antigen Determination

p24-antigen was quantified by ELISA (Murex HIV antigen Mab, Abbott) as described by Garcia *et al*., 2011 [1].

### MT-2 Cell Line

MT-2 cell line was obtained from the AIDS Research and Reference Reagent Program. MT-2 cells are human T-cell leukemia virus type I-transformed lymphoblastoid cell line. HIV-1 strains IIIB is characteristic of syncytia formation when it infects MT-2 cells. The *in vitro* 50% cell culture infectious dose (CCID_50_) per milliliter for virus on MT-2 cells was determined by endpoint dilution analysis (titer of HIV) by Reed & Muench method.

### Viral Stock

HIV-1 strain IIIB (HIV IIIB) was obtained from the AIDS Research and Reference Reagent Program. An HIV IIIB strain was replicated in MT-2 cells, and the viral stock enriched in viral particles was obtained by centrifugation (23,500 g at 4°C during 60 minutes) of supernatants of MT-2 cells. Viruses quantification was carried out by end-point dilution assay in MT-2 cells culture (Cell Culture Infectious Dose 50%, CCID_50_), p24-antigen/ml and viral load (copies HIV-RNA/ml) quantification.

### Viral Capture Assay

4.10^8^ erythrocytes were incubated with 60 pg p24-antigen of particulate viral stock during 30 minutes at 37°C, at 40% hematocrit. After centrifugation at 470 g for 5 minutes the supernatant was recovered and p24-antigen was determined. Capture of HIV by erythrocytes was determined by difference between p24-antigen measurement before and after erythrocyte incubation, with a correction that considered the liquid contained in the erythrocyte fraction. Results were expressed as percentage of HIV capture capacity by erythrocytes.

Previously, in order to choose the best procedure to determine HIV capture by erythrocytes, two methods were compared. p24-antigen was measured by: acid elution directly on the erythrocyte fraction as was used in a previous work [1] and by difference as was mentioned above. Linear regression between both methods was made obtaining a R^2^ of 0.933 using 10 samples from HIV-positive individuals. This analysis showed no significant difference between both methods.

In order to rule out the possibility that the presence of antibodies in the supernatant of the incubation (erythrocyte and HIV) may mask the p24-antigen, previous to p24-antigen measurement, the supernatant was heated for 3 min at 98°C (in order to disaggregate possible immune complexes). Differences less than 8% were observed in 10 cases between heated and unheated supernatant. IgG anti-HIV antibodies were not detected in the supernatant of the last wash of erythrocytes, after viral capture assay.

To check if the p24-antigen measured in supernatants after incubation (erythrocytes and HIV) did not come from erythrocytes of HIV-positive individuals, viral capture assay was made in erythrocytes without virus addition. p24-antigen was measured in the supernatant after 30 minutes of incubation at 37°C and p24-antigen was not detected.

### Inhibition of Viral Capture Assay

Previous to the viral capture assay, erythrocytes from HIV-positive individuals were incubated 30 minutes at 37°C with 7.7 µg/ml of HIV-1 gp120 glycoprotein. The viral capture assay was performed after the washing of erythrocytes. The recombinant gp120 used were gp120 IIIB (Protein Sciences and NIH AIDS Research and Reference Reagent Program), gp120 MN and gp120 CM (Protein Sciences). Presented data only shows the results obtained for gp120 IIIB, although similar results were obtained with the other two variants (data not shown).

### Infectivity After Treatment with Antibodies and Complement and Viral Capture

Plasma dilutions from HIV-positive individuals as source of HIV-specific antibodies (Ig) were inactivated 45 minutes at 56°C. 100 µl of plasma from each individual was incubated with 10,000 CCID50 of CXCR4-tropic strains of HIV IIIB (HIV) for 30 minutes at 37°C in presence of: normal human serum as source of complement (C) (HIV + Ig + C) or in presence of inactivated normal human serum 45 minutes at 56°C (Ci) (HIV + Ig + Ci). An aliquot of each of these samples were exposed to 2.5 10^8^ erythrocytes from HIV-negative individuals (E) 30 minutes at 37°C (viral capture) (HIV-Ig-C +E or HIV-Ig-Ci + E). After incubation, samples were centrifuged and the supernatants collected. Then, MT-2 cells were infected with different dilutions of these supernatants. After 1 hour, cultures were washed 3 times and supplemented with medium. Cultures were incubated for 7 to 10 days and assayed for syncytium formation. Neutralization titers were calculated, expressed as the inverse of the highest dilution that is able to inhibit syncytium formation (loss of infectivity) in MT-2 cells.

Virus alone without treatment, virus with complement and without antibodies and virus with inactivated complement without antibodies were used as controls. Moreover, the presence of erythrocytes alone reduced the infectivity of the free virus available and that effect was taking into account for the calculation of neutralization titers.

### Titers of HIV Infectivity after Viral Capture

2.5 10^8^ erythrocytes from HIV-positive or negative individuals were incubated with 10,000 CCID50 of CXCR4-tropic strains of HIV IIIB (HIV) for 30 minutes at 37°C. After incubation, the samples were centrifuged and the supernatant collected. The infectivity remaining in the supernatants was measured by end-point dilution assay in MT-2 cells culture (titers of infectious HIV).

### Statistical Analysis

Fisher’s Exact Test was used to calculate significance in contingency tables. Nonparametric methods were employed for testing group differences, Mann-Whitney U test and Kruskal-Wallis test for unpaired and Wilcoxon signed-rank test for paired. Correlation analysis was performed using Spearman’s rank correlation. All tests of significance were two-tailed and the level of significance was set at 0.05. In order to show statistical difference between means of HIV capture by erythrocytes of HIV-positive and HIV-negative individuals t test was used.

Two-way ANOVA with random effects and repeated measures analysis were used in the inhibition capture of HIV by erythrocytes assays. For the former, treatment with soluble gp-120 was set as a fixed factor and subjects under analysis were considered a random factor. In this analysis a significant p-value for the fixed factor means an association of the variable with the treatment with gp-120 and a significant p-value for the random factor means a significant variation of the association among individuals under study. The repeated measures analysis was applied when patients where further classified according to the presence of antibodies against gp120 attached to erythrocytes, considering this characteristic as the fixed factor and each of the measures of percentage of capture with and without soluble gp-120 as repeated measures over the same group of patients. For this analysis, assumption of equality of covariance matrices were assessed by Box’s Test, assumption of sphericity was assessed by Mauchly’s Test, and assumption of equality of error variances was assessed by Levene’s Test.

## Supporting Information

Figure S1 **Selective IgG anti-HIV adherence to erythrocytes.** Pattern of immunoglobulin G anti-HIV (IgG anti-HIV) determined by western blot in plasma of patients (IgG anti-HIV-P), plasma diluted at the same concentration than 100x concentrated IgG present in purified erythrocytes (IgG anti-HIV-Pd), purified erythrocytes (IgG anti-HIV-E), and IgG anti-HIV-E 100x concentrated (IgG anti-HIV-Ec). Identical or similar (difference in the presence of one to three IgG anti-HIV) patterns were observed between IgG anti-HIV-E and IgG anti-HIV-Ec. On the other hand, all IgG anti-HIV evaluated were found in plasma while only one or two were observed in diluted plasmas. Notably, we could not find an association between IgG anti-HIV pattern in erythrocytes and diluted plasmas suggesting selective IgG anti-HIV binding to erythrocyte membrane. Representative IgG anti-HIV patterns from two of the eight HIV-positive individuals evaluated are shown.
(TIF

Table S1 **Detection of IgGs anti-HIV, plasma viral load, p24-antigen and HIV capture by erythrocytes in HIV-positive individuals.**
(DOC)
