# Supplementary figures and images for: Presence of IgG Anti-gp160/120 Antibodies Confers Higher HIV Capture Capacity to Erythrocytes from HIV-Positive Individuals
Source: PLoS One. 2012 Sep 25;7(9):e45808. doi: 10.1371/journal.pone.0045808 (PMC3458065; doi:10.1371/journal.pone.0045808)

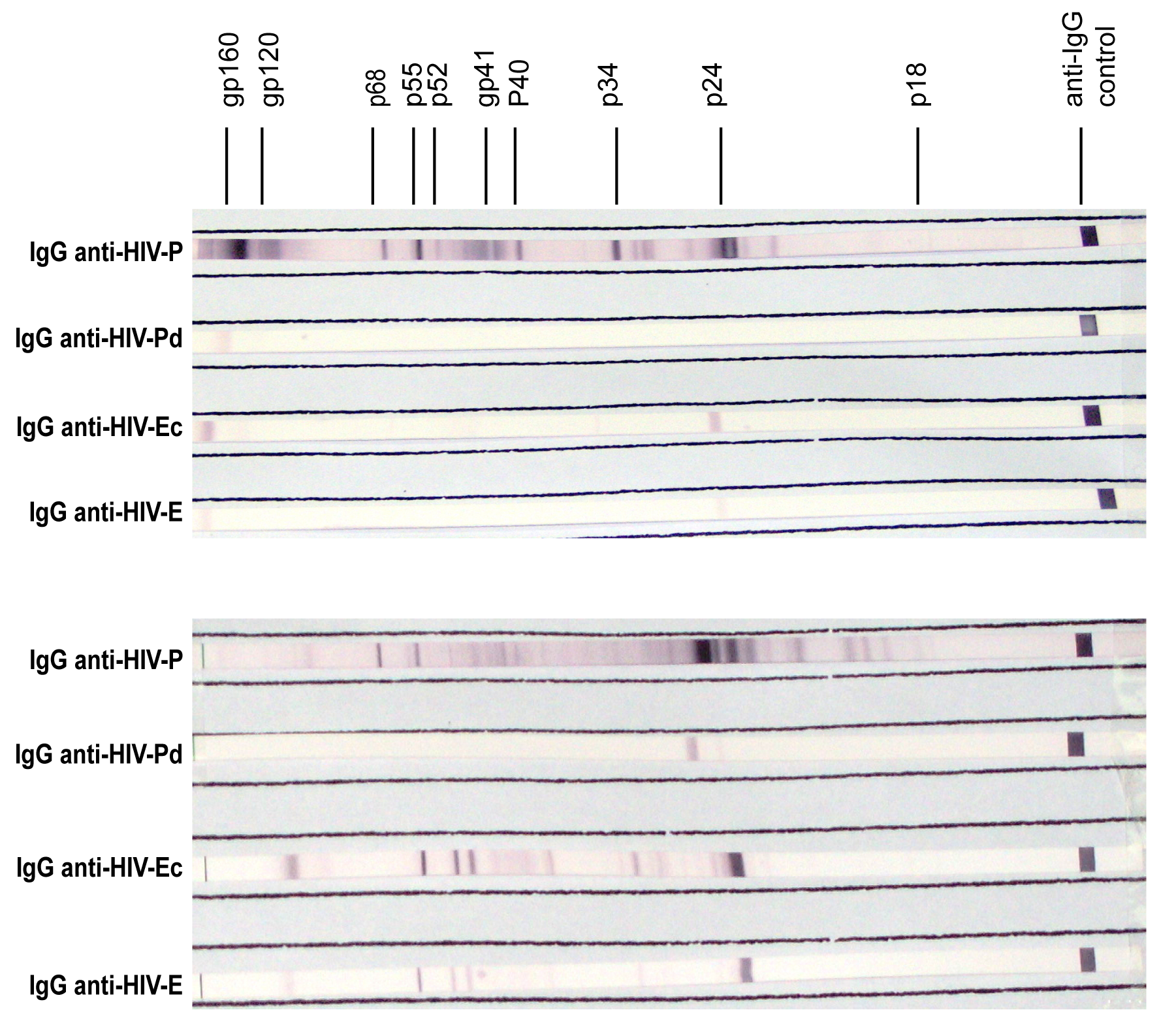

Supplement: Figure S1 — Selective IgG anti-HIV adherence to erythrocytes. Pattern of immunoglobulin G anti-HIV (IgG anti-HIV) determined by western blot in plasma of patients (IgG anti-HIV-P), plasma diluted at the same concentration than 100x concentrated IgG present in purified erythrocytes (IgG anti-HIV-Pd), purified erythrocytes (IgG anti-HIV-E), and IgG anti-HIV-E 100x concentrated (IgG anti-HIV-Ec). Identical or similar (difference in the presence of one to three IgG anti-HIV) patterns were observed between IgG anti-HIV-E and IgG anti-HIV-Ec. On the other hand, all IgG anti-HIV evaluated were found in plasma while only one or two were observed in diluted plasmas. Notably, we could not find an association between IgG anti-HIV pattern in erythrocytes and diluted plasmas suggesting selective IgG anti-HIV binding to erythrocyte membrane. Representative IgG anti-HIV patterns from two of the eight HIV-positive individuals evaluated are shown. (TIF [file pone.0045808.s001.tif]
