# Supplementary material for: Presence of IgG Anti-gp160/120 Antibodies Confers Higher HIV Capture Capacity to Erythrocytes from HIV-Positive Individuals
Source: PLoS One. 2012 Sep 25;7(9):e45808. doi: 10.1371/journal.pone.0045808 (PMC3458065; doi:10.1371/journal.pone.0045808)
Supplement: Table S1 — Detection of IgGs anti-HIV, plasma viral load, p24-antigen and HIV capture by erythrocytes in HIV-positive individuals. (DOC) [file pone.0045808.s002.doc]

**Table S1- *Detection of IgGs anti-HIV, plasma viral load, p24-antigen and HIV capture by erythrocytes in HIV-positive individuals.***

**IgG anti-HIV-E**

| **HIV**  **positive**  **individuals** | **pVL**  **(copies/ml)** | **(%) capture of HIV by erythrocytes** | **Ag-E**  **(pg/ml)** | | **gp160** | **gp120** | **p68** | **p55** | **p52** | **gp41** | **p40** | **p34** | **p24** | **p18** |
| --- | --- | --- | --- | --- | --- | --- | --- | --- | --- | --- | --- | --- | --- | --- |
| **1** | <50 | 55.2 | ND |  | |  |  |  |  |  |  |  |  |  |
| **2** | <50 | 41.5 | ND |  | |  |  |  |  |  |  |  |  |  |
| **3** | <50 | 1.1 | ND |  | |  |  |  |  |  |  |  |  |  |
| **4** | <50 | 38.0 | ND |  | |  |  |  |  |  |  |  |  |  |
| **5** | <50 | 0.0 | ND |  | |  |  |  |  |  |  |  | x |  |
| **6** | <50 | 13.4 | ND |  | |  |  |  |  |  |  |  |  |  |
| **7** | <50 | 1.0 | ND |  | |  |  |  |  |  |  |  |  |  |
| **8** | <50 | 9.0 | ND |  | |  |  |  |  |  |  |  |  |  |
| **9** | <50 | 8.3 | ND |  | |  |  |  |  |  |  |  | x |  |
| **10** | <50 | 58.1 | ND | x | |  |  |  |  |  |  |  | x |  |
| **11** | <50 | 8.8 | ND |  | |  |  |  |  |  |  |  |  |  |
| **12** | <50 | 36.1 | ND | x | |  |  |  |  |  |  |  |  |  |
| **13** | <50 | 63.1 | ND |  | |  |  |  |  |  |  |  |  |  |
| **14** | <50 | 75.9 | ND |  | |  |  |  |  |  |  |  |  |  |
| **15** | <50 | 86.6 | ND | x | |  |  |  |  |  |  |  |  |  |
| **16** | <50 | 70.3 | ND | x | |  |  |  |  |  |  |  | x |  |
| **17** | <50 | 65.0 | ND | x | |  |  |  |  |  |  |  | x |  |
| **18** | <50 | ND | ND | x | |  |  |  |  |  |  | x | x |  |
| **19** | <50 | ND | ND |  | |  |  |  |  |  |  |  |  |  |
| **20** | <50 | ND | ND |  | |  |  |  |  |  |  |  | x |  |
| **21** | <50 | ND | ND | x | |  |  |  |  |  |  |  |  |  |
| **22** | <50 | ND | ND | x | |  |  |  |  |  |  |  |  |  |
| **23** | <50 | ND | 55 | x | |  |  |  |  |  |  |  | x |  |
| **24** | <50 | ND | ND | x | |  |  |  |  |  |  |  | x |  |
| **25** | <50 | ND | 109.2 | x | |  |  |  |  |  |  |  |  |  |
| **26** | 68 | 0.0 | ND | x | |  |  |  |  |  |  |  |  |  |
| **27** | 70 | 12.9 | ND | x | |  |  |  |  |  |  |  |  |  |
| **28** | 99 | 0.0 | ND |  | |  |  |  |  |  |  |  |  |  |
| **29** | 118 | 3.0 | ND |  | |  |  |  |  |  |  |  |  |  |
| **30** | 138 | 6.3 | ND |  | |  |  |  |  |  |  |  | x |  |
| **31** | 191 | 40.7 | ND | x | | x | x | x |  | x |  |  | x |  |
| **32** | 1,406 | ND | 107.6 | x | |  | x | x | x | x |  | x | x |  |
| **33** | 2,023 | 10.9 | ND |  | |  |  |  |  |  |  |  | x |  |
| **34** | 2,782 | 23.2 | ND | x | |  |  |  |  |  |  | x | x |  |
| **35** | 3,196 | 29.0 | ND | x | |  | x |  |  |  |  |  |  |  |
| **36** | 3,771 | ND | 59 | x | |  | x |  |  |  |  |  | x |  |
| **37** | 4,858 | 15.3 | ND | x | |  | x |  | x | x | x | x |  |  |
| **38** | 5,464 | 38.0 | ND | x | |  | x | x |  |  |  | x |  |  |
| **39** | 6,470 | ND | ND | x | | x |  |  |  | x |  |  | x |  |
| **40** | 7,307 | 6.8 | ND |  | |  |  |  |  |  |  |  |  |  |
| **41** | 10,310 | ND | 64 | x | | x | x |  |  | x |  | x |  |  |
| **42** | 10,390 | 54.9 | ND | x | | x | x | x |  | x |  | x |  |  |
| **43** | 10,751 | ND | ND | x | |  | x | x |  |  |  | x | x |  |
| **44** | 10,791 | 65.0 | ND | x | |  | x | x |  |  |  | x | x |  |
| **45** | 12,406 | 95.1 | ND | x | |  | x |  |  |  |  | x | x |  |
| **46** | 12,426 | ND | ND | x | |  | x |  |  |  |  | x | x |  |
| **47** | 13,053 | 100 | ND | x | | x | x | x | x | x | x | x | x |  |
| **48** | 13,817 | 0.0 | ND |  | |  |  |  |  |  |  |  |  |  |
| **49** | 17,969 | ND | 53 | x | |  |  |  |  |  |  |  | x |  |
| **50** | 18,799 | 0.0 | ND | x | |  |  |  |  |  |  |  |  |  |
| **51** | 22,217 | ND | 116.4 | x | |  |  |  |  |  |  | x |  |  |
| **52** | 22,333 | ND | 33 |  | |  | x |  |  |  |  | x |  |  |
| **53** | 23,743 | ND | ND | x | |  | x | x | x |  |  | x | x |  |
| **54** | 29,027 | 100 | ND | x | |  | x | x | x |  | x | x | x |  |
| **55** | 30,165 | 0.0 | ND |  | |  |  |  |  |  |  |  | x |  |
| **56** | 34,682 | 21.7 | ND |  | |  |  |  |  |  |  |  |  |  |
| **57** | 37,364 | 27.5 | ND |  | |  |  |  |  |  |  |  |  |  |
| **58** | 45,594 | ND | 96 | x | |  |  |  |  |  |  | x |  |  |
| **59** | 45,637 | 65.0 | ND | x | | x |  |  |  | x |  |  | x |  |
| **60** | 49,851 | 69.7 | ND | x | |  |  |  |  |  |  | x |  |  |
| **61** | 49,871 | ND | 98 | x | |  |  |  |  |  |  | x |  |  |
| **62** | 54,387 | 69.0 | ND | x | | x |  |  |  | x |  |  | x |  |
| **63** | 62,746 | ND | ND | x | |  | x |  | x | x |  | x | x | x |
| **64** | 75,637 | ND | >152 | x | |  |  |  |  |  |  |  | x |  |
| **65** | 79.472 | 31.0 | ND | x | |  |  | x |  |  |  |  | x |  |
| **66** | 206,242 | 1.4 | ND |  | |  |  |  |  |  |  |  | x |  |
| **67** | 264,670 | ND | ND | x | |  | x | x | x | x | x | x | x |  |
| **68** | 273,689 | 11.4 | ND | x | | x |  |  |  | x |  |  | x |  |
| **69** | 282,301 | 0.6 | ND |  | |  |  |  |  |  |  |  | x |  |
| **70** | 316,443 | ND | 110.8 | x | |  |  |  |  |  |  |  | x |  |
| **71** | 335,366 | ND | ND | x | | x |  |  |  | x |  |  |  |  |
| **72** | 348,147 | ND | ND | x | | x | x | x | x | x |  | x | x |  |
| **73** | 348,167 | 20.8 | ND | x | | x | x | x |  | x |  | x | x |  |
| **74** | >500,000 | 73.2 | ND | x | |  |  |  |  |  |  |  |  |  |
| **75** | >500,000 | ND | ND | x | |  |  |  |  |  |  |  |  |  |
|  | **Mean** | **33.82** |  |  | |  |  |  |  |  |  |  |  |  |

**pVL:** plasma viral load at the moment of the study.

**(%) Capture of HIV by erythrocytes:** percent of p24-antigen adsorbed to erythrocytes.

**Ag-E (pg/ml):** pg erythrocytes-associated p24-antigen per milliliter of purified erythrocytes.

**IgG anti-HIV-E:** immunoglobulin G anti-HIV in erythrocytes from HIV-positive individuals.

**ND:** no determined.
